# Accuracy of an Electronic Apex Locator (EAL) in Measuring the Working Length of Root Canals in Comparison With Radiographs: An In Vivo Study

**DOI:** 10.7759/cureus.60283

**Published:** 2024-05-14

**Authors:** Esha Bhagat, Yoshaskam Agnihotri, Abhisek Das, Sambarta Das, Swagat Panda, Sudeepta Hota

**Affiliations:** 1 Conservative Dentistry and Endodontics, Hi-Tech Dental College and Hospital, Bhubaneswar, IND

**Keywords:** working length, minor diameter, major diameter, apical foramen, apex locators

## Abstract

Introduction: Traditionally, radiographs were used to assess the working length of root canals. However, the use of Electronic Apex Locators (EAL) may be a non-invasive alternative. This study aimed to evaluate the accuracy of the electronic apex locator (EAL) compared to radiographic measurements in determining the working length of root canals.

Method: The study assessed the performance of EALs in different scenarios, including the presence of blood and pulp tissue, the use of ethylenediaminetetraacetic acid (EDTA), sodium hypochlorite (NaOCl) as an irrigant, and post-instrumentation with normal saline. An electronic apex locator (DTE DPEX-V; Woodpecker, China) was used alongside traditional radiographs to measure the working length in 144 root canal treatments. Bland-Altman analysis was used to compare the measurements between EAL and radiographs using Jamovi software, v2.4.8 (https://www.jamovi.org/).

Result: The findings revealed strong correlations between EAL and radiographic measurements across all testing environments, with Pearson's correlation coefficients ranging from 0.944 to 0.998. The Bland-Altman analysis suggests bias; the average difference was negative and close to zero (Pulp: -0.354, EDTA: -0.0972, NaOCl: -0.0382, Saline: -0.0139) when EAL measurements were compared to radiographic measurements.

Conclusion: The working length measurement of root canals using an electronic apex locator such as DTE DPEX-V is comparable to traditional radiographic measurements. The use of irrigants improves the measurement. The EAL has the potential to be an alternative to the invasive radiograph for root canal length measurement.

## Introduction

Determining the accurate working length of the root canal is a cornerstone in endodontic treatment, directly influencing its success and durability. Traditionally, radiographs have been the gold standard for this purpose, offering a visual representation of the canal's length and anatomy. However, the advent of electronic apex locators (EALs) has revolutionized this aspect of endodontic practice [1]. These devices provide clinicians with a means to measure working lengths, promising to mitigate the limitations associated with radiographic methods, such as exposure to radiation, the challenge of interpreting two-dimensional representations of three-dimensional structures, and the variability introduced by anatomical differences [2].

EALs represent a significant technological advancement over traditional methods like radiographs and tactile feedback, which often lack precision due to distortions and subjective interpretations [3]. EALs measure electrical impedance within the canal, reducing inconsistencies and avoiding radiation exposure. This leads to more accurate measurements and enhances patient safety, making EALs especially beneficial in complex cases with irregular canal morphology [4].

For successful root canal treatments, the precise determination of the working length is critical because it ensures thorough cleaning, disinfection, and appropriate sealing of the root canal system, ultimately influencing treatment outcomes and patient satisfaction [5]. While conventional radiographs have long served as the benchmark for accuracy, the introduction of EALs offers a potentially more accurate and efficient alternative [1,4]. Other techniques used for determining working length, such as digital tactile sense, apical periodontal sensitivity, and paper point measurements, exist but are less reliable and susceptible to intra-subject variation [6].

Correctly determining the working length, defined as “the distance from a coronal reference point to the point at which canal preparation and obturation should terminate”, is critical to the outcome of endodontic therapy [5]. Accurate determination of the working length establishes the apical endpoint for instrumentation and obturation, which is vital for maintaining the integrity of the apical constriction [6,7]. Precise measurement of the working length prevents under-instrumentation, which might leave residual tissues and debris in the apex, as well as over-instrumentation that could cause patient discomfort, damage periapical tissues, or potentially lead to infection or cyst development due to the placement of irritating materials beyond the apex [8].

While studies assessing the accuracy of EALs remain limited in Indian settings [9-11], a pressing need for further exploration is a requirement. There is a gap in research regarding comparisons of EAL measurements in different clinical settings, such as narrow and wide canals, and the influence of various irrigants. The objective of this study is to assess the accuracy of apex locator (DTE DPEX- V; Woodpecker, China) readings compared to routinely used radiographic measurements under various clinical conditions, including the presence of blood and pulp tissue, exposure to 17% ethylenediaminetetraacetic acid (EDTA) and 3% sodium hypochlorite (NaOCl), and post-instrumentation with normal saline.

## Materials and methods

The present study was conducted in the Department of Conservative Dentistry and Endodontics, Hi-Tech Dental College and Hospital, Bhubaneswar, Odisha, India, after gaining ethical clearance from the Institutional Ethical Committee with reference no. HMCH/IEC/2022/173 dated on 20th May, 2022.

Seventy-two posterior teeth, spanning both the maxillary and mandibular arches, were carefully selected from patients aged 18 to 35 years. The inclusion criteria were to admit only teeth with completely formed apices devoid of carious lesions, coronal restorations, or structural cracks. Teeth exhibiting resorption defects, open apices, canal obliteration, or periapical pathologies were identified and excluded from the study after a thorough radiographic evaluation.

After obtaining informed consent from the participants, local anesthesia was administered using LIGNOX 2% A (which comprised of lignocaine and adrenaline in 1:80000 ratio; Indoco Remedies Limited, Navi Mumbai, India), and each tooth was isolated using a rubber dam to establish a sterile field for intervention. The rubber dam utilized was of COLTENE brand, sized 6” x 6” (Whaledent Pvt. Ltd., Mumbai, India) and composed of latex. Access cavities were prepared precisely using MANI Endo Access bur (EA 10) (Mani, Inc., Tochigi, Japan) with the following characteristics: total length of 22.0 mm, working portion length of 11.0 mm, and a maximum diameter of 1.9 mm, ensuring minimal disruption to the surrounding tooth structure. The teeth were then evaluated with an advanced EAL, DTE DPEX-V (Woodpecker, China).

The DTE DPEX-V, a fifth-generation apex locator, was used for accurate working length determination. Equipped with Anti-Interference and based on advanced multiple-frequency network impedance measurement technology and automatic calibration, it ensures stability and precision in challenging conditions. Consistent across tooth types, it offers enhanced accuracy and ease of use with a multi-colour liquid crystal display and alarm function [12].

The investigative protocol subjected each tooth to four distinct clinical conditions: the presence of pulp tissue, immersion in 17% EDTA solution, exposure to 3% NaOCl solution, and saturation in normal saline. The operational parameters of the apex locators were standardized to a zero reading. The determination of working length was facilitated through the utilization of K-files (#10, #15 and #20 K files were used with a taper of 2%) in conjunction with radiographic validation. The data thus acquired from the apex locators under varying clinical scenarios were measured and compared with the radiographic measurements of working length, fostering a comprehensive evaluation of device accuracy under diverse endodontic conditions.

The root canal length measurements were summarized using mean and standard deviation. The measurements by X-ray and EAL were compared using correlation statistics. The accuracy of EAL measurements was assessed using Bland-Altman analysis to see if both measurements were in agreement. The statistical analysis was done using Jamovi software, v.2.4.8 (https://www.jamovi.org/).

## Results

Table 1 presents the results of various testing conditions relative to radiographic measurements. It provides information on the number of observations (n), mean values, standard deviations, and correlations with radiographic measurements for different testing environments. The study included 144 observations from wide and narrow canals of posterior premolars and molars. The mean root canal length, as measured in various testing environments, was relatively similar, ranging from 18.069 for pulp to 18.424 for X-ray.

**Table 1:** Correlation between the EAL and X-ray measurements of root canal working length *The correlation is significant at p < 0.001 level, r = Pearsons' correlation coefficient EAL: electronic apex locator (DTE DPEX-V; Woodpecker, China), EDTA: Ethylenediaminetetraacetic acid, NaOCl: Sodium hypochlorite.

Testing Environment	Mean	Standard Deviation	Correlation with Radiographic Measurement (r)
Pulp Tissue (n=144)	18.069	1.3296	0.944*
EDTA (n=144)	18.326	1.3527	0.982*
NaOCl (n=144)	18.385	1.3788	0.994*
Saline (n=144)	18.41	1.3685	0.996 *
X-ray (n=144)	18.424	1.354	1

All the EAL measurements in various testing environments had a very strong correlation with X-ray measurements. The correlation was highly significant (p<0.001). The saline environment exhibited the highest Pearson correlation coefficient (r=0.996), followed by NaOCl (r=0.994), EDTA (r=0.982), and pulp (0.994). The Pulp Tissue environment had the lowest correlation among the tested environments at 0.944, still a very strong positive relationship.

Table 2 presents the results of various testing environments for working length determination, stratified by root canal type (narrow canal and wide canal), and compared to radiographic measurements. Across all testing environments, the wide canal group consistently exhibited higher mean values (ranging from 18.9 to 19.3) compared to the narrow canal group (ranging from 17.4 to 17.7). The correlations with radiographic measurements were very strong (r > 0.9) and highly significant irrespective of the type of root canal. However, EAL measurements in wide canals had a higher Pearson correlation coefficient than narrow canal measurements.

**Table 2:** Correlation between the EAL and X-ray measurements of root canal working length stratified by root canal type *The correlation is significant at p < 0.001 level EAL: electronic apex locator (DTE DPEX-V; Woodpecker, China), EDTA: Ethylenediaminetetraacetic acid, NaOCl: sodium hypochlorite.

Testing environment	Root canal type	Mean	Standard deviation	Correlation with radiographic measurement (r)
Pulp Tissue	Narrow Canal (n=80)	17.4	1.05	0.906*
	Wide Canal (n=64)	18.9	1.15	0.928*
EDTA	Narrow Canal (n=80)	17.6	1.01	0.954*
	Wide Canal (n=64)	19.3	1.15	0.991*
NaOCl	Narrow Canal(n=80)	17.7	1.03	0.980*
	Wide Canal (n=64)	19.3	1.19	0.998*
Saline	Narrow Canal (n=80)	17.7	1.04	0.989*
	Wide Canal (n=64)	19.3	1.19	0.998*
X-ray	Narrow Canal (n=80)	17.7	1.02	1.000
	Wide Canal (n=64)	19.3	1.21	1.000

Table 3 presents the results of a Bland-Altman analysis, which assesses the agreement between radiographic measurements and an EAL (DTE DPEX-V) under various irrigant conditions. For the Pulp Tissue or no irrigant condition, the bias (mean of differences) was -0.354, with limits of agreement ranging from -1.236 to 0.528, indicating a potential underestimation by the EAL compared to radiographic measurements. With the EDTA irrigant, the bias was reduced to -0.0972, and the limits of agreement were narrower (-0.6061 to 0.4116), suggesting better agreement. The NaOCl irrigant showed a smaller bias of -0.0382 and the narrowest limits of agreement (-0.3466 to 0.2702). Lastly, the saline irrigant condition had a bias of -0.0139 and limits of agreement from -0.2441 to 0.2163, also demonstrating good agreement with radiographic measurements.

**Table 3:** Agreement between EAL and radiographic measurements using Bland-Altman analysis EAL: electronic apex locator (DTE DPEX-V; Woodpecker, China), EDTA: Ethylenediaminetetraacetic acid, NaOCl: sodium hypochlorite.

Sl No	Irrigant	Estimates	Estimate	95% Confidence Interval	
				Lower	Upper
1	No Irrigant (Measurement with Pulp Tissue)	Bias	-0.354	-0.428	-0.28
		Lower limit of agreement	-1.236	-1.363	-1.109
		Upper limit of agreement	0.528	0.401	0.654
2	Irrigant (EDTA)	Bias	-0.0972	-0.14	-0.0545
		Lower limit of agreement	-0.6061	-0.679	-0.5328
		Upper limit of agreement	0.4116	0.338	0.4849
3	Irrigant (NaOCl)	Bias	-0.0382	-0.0641	-0.0123
		Lower limit of agreement	-0.3466	-0.391	-0.3022
		Upper limit of agreement	0.2702	0.2258	0.3146
4	Irrigant (Saline)	Bias	-0.0139	-0.0332	0.00546
		Lower limit of agreement	-0.2441	-0.2772	-0.21093
		Upper limit of agreement	0.2163	0.1832	0.24943

**Figure 1:**
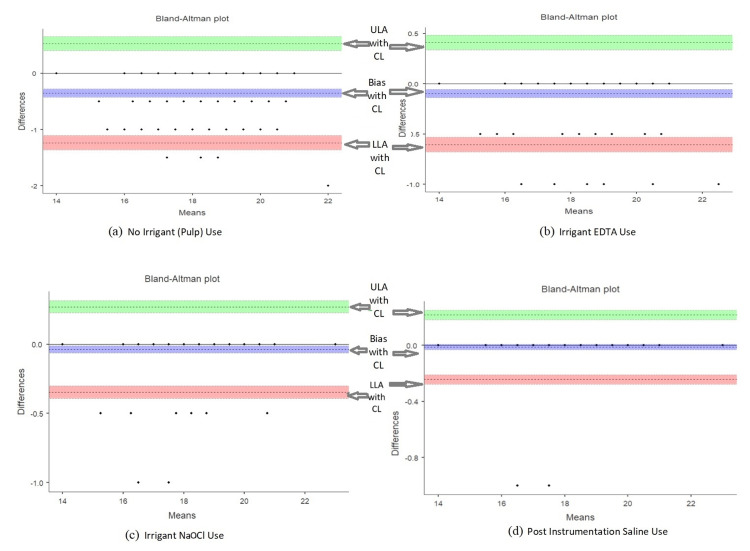
Bland-Altman plots ULA: Upper limit of agreement, LLA: Lower limit of agreement, CL: Confidence limit.

The Bland-Altman plot helps visualization of agreement between two methods of measuring the working length of root canals under various testing conditions (Figure 1). Measurement with pulp tissue, the plot suggests the data points are clustered around the mean difference line, which is below zero, indicating an underestimation bias. The limits of agreement are relatively wide, suggesting differences between the two methods. Measurement with EDTA irrigant, the plot suggests, the data points are more tightly clustered around the mean difference line, which is close to zero, indicating a minimal bias. The limits of agreement are narrower compared to Pulp Tissue, suggesting better agreement between the methods.

Measurement with NaOCl irrigant, the plot suggests, the data points are clustered very tightly around the mean difference line, which is closer to zero indicating minimal bias. The limits of agreement are the narrowest, suggesting minimal differences between the two methods. In measurement with saline irrigant, the data points are also tightly clustered around the mean difference line, which is slightly above zero but close to it indicating minimal bias. The limits of agreement are slightly narrower than in the NaOCl. The limits of agreement are narrowest among all four environments, suggesting the smallest range of differences between the two measurement methods.

## Discussion

This study evaluated the accuracy of EAL compared to radiographic measurements in determining working length under various clinical conditions. The accuracy of the working length assessment directly impacts the success and longevity of root canal treatments. While traditional radiographs have served as the standard method, the introduction of EAL offers a promising alternative, potentially overcoming the limitations associated with radiographic techniques [13]. By assessing the performance of apex locators under different conditions, including the presence of blood and pulp tissue, exposure to irrigants such as EDTA and NaOCl, and post-instrumentation with normal saline, this study aims to provide valuable insights into the reliability and effectiveness of EALs in diverse clinical scenarios.

The apical constriction serves as the appropriate endpoint for root canal therapy, significantly influencing treatment prognosis [14]. Accurately pinpointing the apical constrictions is vital; exceeding it risks overfilling and potential complications while falling short leaves behind harmful bacteria and tissue [15]. Thus, the apex locator's precision in identifying the apical constrictions is pivotal for effective treatment outcomes. A correct determination of working length has a substantial impact on endodontic treatment prognosis. More precise determination of the working length is now feasible, due to the latest generation of apex locators. However, clinicians are gravely concerned about the accuracy of these apex locators. Radiography would not be enough to identify the working length; the patient would be exposed to excessive radiation throughout the root canal procedure, and an excessive number of radiographic photographs would be acquired. The EALs operate based on electrical impedance principles rather than the biological properties of the tissue [16-18], which could potentially offer advantages over radiographic methods in certain scenarios like severe curvatures, calcifications, or atypical anatomical variations.

Jha et al., in 2021 [19], evaluated the accuracy of the apex locator in the presence of different irrigating solutions, including 3% NaOCl, 5% NaOCl, 2% chlorhexidine, and 17% EDTA. The study found that there were no statistically significant differences between the gold standard and electronic apex locator lengths in any of the groups. However, the study does not directly compare the accuracy of the apex locator in the presence of pulp tissue remnants and irrigating solutions. The results in this study demonstrated that the apex locator exhibited very strong correlations with radiographic measurements across all testing environments, ranging from 0.944 for measurements with pulp tissue to 0.998 for measurements after NaOCl and saline irrigation. This aligns with the findings of Jha et al., suggesting that irrigating solutions generally improve the accuracy of apex locators compared to environments with organic debris. The findings reveal that the accuracy of the apex locator improves significantly when used in conjunction with irrigating solutions, particularly NaOCl and saline, as opposed to measurements taken in the presence of pulp tissue remnants. The present study found that NaOCl and saline outperformed EDTA in terms of agreement with radiographic measurements, which could be attributed to their distinct mechanisms of action and their ability to remove organic and inorganic debris more effectively. These findings underscore the importance of proper canal preparation and irrigation protocols when using electronic apex locators to optimize their accuracy [4,20].

The Bland-Altman analysis further corroborated these results, illustrating the narrowest limits of agreement and minimal bias between the apex locator and radiographic measurements when NaOCl and saline irrigants were used. This suggests that the removal of organic debris and the establishment of a more conducive environment within the root canal system enhanced the precision of the apex locator's measurements. These findings further emphasize the importance of effective irrigation protocols in optimizing the accuracy of electronic apex locators.

This study’s findings underscore the importance of proper canal preparation and irrigation protocols when using electronic apex locators to optimize their accuracy. The superiority of NaOCl and saline irrigants over EDTA in improving the agreement between the apex locator and radiographic measurements could be attributed to their distinct mechanisms of action and their ability to remove organic and inorganic debris more effectively. This highlights the crucial role of selecting the appropriate irrigating solution based on its ability to facilitate accurate apex locator readings, thereby contributing to the overall success of endodontic treatment.

Lastly, the study's findings highlight the potential influence of canal anatomy on the accuracy of apex locators. Across all testing environments, the measurements obtained in wide canals consistently exhibited stronger correlations with radiographic measurements compared to narrow canals. This observation aligns with previous research suggesting that the accuracy of apex locators may be influenced by factors such as canal curvature, calcifications, and the presence of apical constrictions [21]. Understanding these anatomical variations can aid clinicians in interpreting apex locator readings more effectively and making informed treatment decisions.

The current study has several limitations. First, the accuracy of apex locator readings may be compromised in cases involving periapical pathologies, such as abscesses or purulent discharge, which could potentially skew the results. Second, the study did not include any non-vital teeth or cases with necrotic pulpal tissue, limiting the generalizability of the findings to this subset of endodontic cases. Third, the study did not assess the performance of apex locators in teeth with immature apices, a scenario often encountered in clinical practice, leaving questions about the applicability of the results in such situations. Fourth, the current study focused solely on evaluating a single electronic apex locator model, failing to provide a comparative analysis across different commercially available devices, which could vary in their accuracy and performance characteristics. These limitations underscore the need for further research to comprehensively evaluate the reliability and precision of apex locators across a broader range of clinical scenarios and device types.

## Conclusions

This study demonstrates that the accuracy of the electronic apex locator in estimating the working length of root canals is at par with that of X-rays. The performance of the electronic apex locator can be affected by the root canal type and irrigation protocols employed. This study suggests the use of NaOCl irrigant or saline while estimating the root canal working length can improve the accuracy of the measurement with the DTE DPEX-V apex locator. Therefore, the use of an electronic apex locator can be a safe alternative to the invasive X-ray. The findings underscore the importance of meticulous canal preparation and judicious selection of irrigating solutions to optimize debris removal and enhance the accuracy of apex locators. Ultimately, the integration of electronic apex locators with appropriate irrigation regimens offers a reliable and efficient alternative to traditional radiographic methods, facilitating precise working length determination and improving the overall success of endodontic treatments.
